# 27 Years of Catalytic Carbonylative Coupling Reactions in Hungary (1994–2021)

**DOI:** 10.3390/molecules27020460

**Published:** 2022-01-11

**Authors:** Tímea R. Kégl, László T. Mika, Tamás Kégl

**Affiliations:** 1 Department of Inorganic Chemistry and ELKH-PTE Research Group for Selective Chemical Syntheses, University of Pécs, Ifjúság útja 6., H-7624 Pécs, Hungary; trkegl@gamma.ttk.pte.hu; 2 Department of Inorganic Chemistry and János Szentágothai Research Centre, University of Pécs, Ifjúság útja 6., H-7624 Pécs, Hungary; 3 Department of Chemical and Environmental Process Engineering, Budapest University of Technology and Economics, Műegyetem rkp. 3, H-1111 Budapest, Hungary; laszlo.t.mika@edu.bme.hu

**Keywords:** aminocarbonylation, alkoxycarbonylation, hydroxycarbonylation, cabon monoxide, cross-coupling

## Abstract

Palladium-catalyzed carbonylation reactions, in the presence of nucleophiles, serve as very potent tools for the conversion of aryl and alkenyl halides or halide equivalents to carboxylic acid derivatives or to other carbonyl compounds. There are a vast number of applications for the synthesis of simple building blocks as well as for the functionalization of biologically important skeletons. This review covers the history of carbonylative coupling reactions in Hungary between the years 1994 and 2021.

## 1. Introduction

One of the fundamental questions of organic chemistry is the selective formation of new carbon-carbon and carbon-heteroatom bonds [1,2,3]. In the last few decades, metal assisted cross-coupling reactions have gained incredible popularity because of their enormous synthetic potential, their versatility, functional group tolerance, and often excellent chemoselectivity [4].

Carbonylative couplings involve the incorporation of one or two carbon monoxide molecules resulting in carbonyl compounds such as ketones, esters, amides or ketoamides [5,6]. The incoming carbon monoxide molecule usually comes from the CO atmosphere or, in some rare cases, from CO surrogates such as DMF or Mo(CO)_6_.

**Figure 1:**
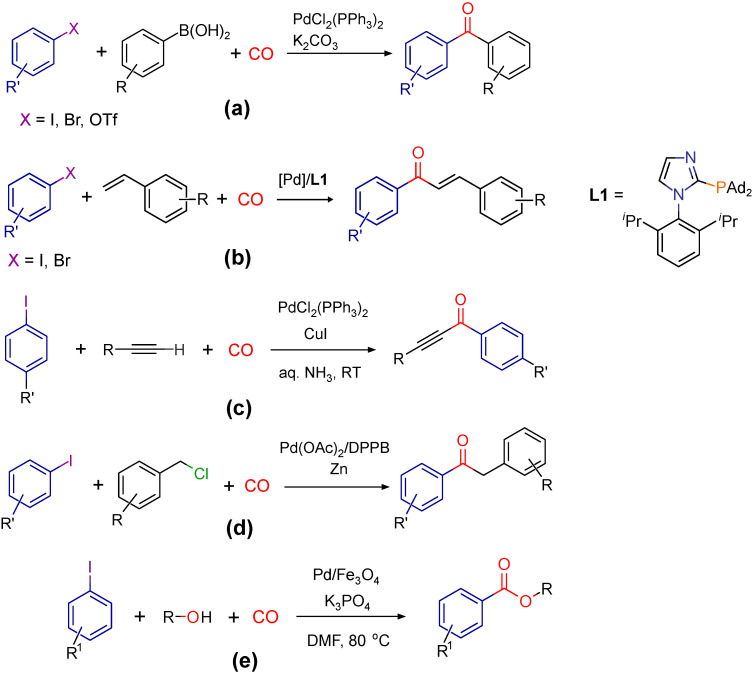
Carbonylative cross-coupling reactions: Carbonylative Suzuki-Miyaura [7] (**a**), Heck [8] (**b**), Sonogashira [9] (**c**), Negishi [10] (**d**) reactions, and the alkoxycarbonylation of aryl iododes [15] (**e**).

Some selective examples for carbonylative coupling are shown in Figure 1. Carbonylative Suzuki reacion is suitable for the preparation of diaromatic ketones from various aril-halides and boronic acids (a) [7]. Beller and co-workers synthesized aromatic enones from iodoarenes and styrene in carbonylative Heck reactions (b) [8]. Mori and co-workers prepared α,β-alkynyl ketones from phenylacetilene and iodoarenes in carbonylative Sonogashira couplings in the presence of copper(I)-iodide, carbon-monoxide and ammonia (c) [9]. A selective example was reported by the Beller-group for a series of carbonylative Negishi reactions aiming at the preparation of 1,2-diaryl-etanones from substituted iodoarenes and benzyl chloride (d) [10]. The aryloxycarbonylation reaction (e) differs from the examples cited above, in which the CO group is formally inserted between a carbon and an oxygen.

**Figure 2:**
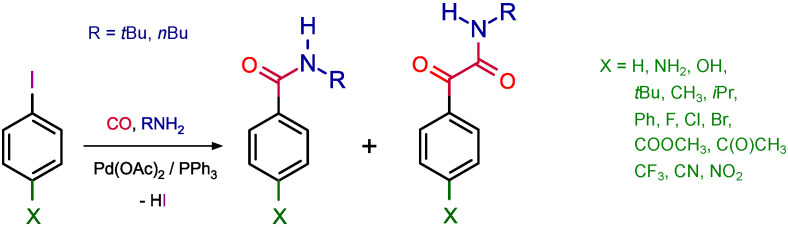
Aminocarbonylation of 4-substituted iodobenzenes [14].

In the presence of *N*-nucleophiles, the formation of a new carbon-carbonyl carbon-nitrogen bonding pattern is possible and this reaction is usually referred as aminocarbonylation. In the seminal works of Yamamoto and co-workers double carbonylation was also reported [11,12,13]. Figure 2 represents an example where 4-substituted iodobenzenes were reacted with primary amines [14]. At atmospehric CO pressure, the formation of amides was predominant, however, the ratio of the double carbonylated products, that is ketoamides, was increased by the increase of the pressure.

To the best of our knowledge, the history of carbonylative coupling reactions in Hungary started back in 1994 when Professor László Kollár and Rita Skoda-Földes vinylated steroidal triflates in the absence and in the presence of carbon monoxide [16] (vide infra) at the University of Veszprém (University of Pannonia, since 2006). Professor Kollár had impressive experience in the field of catalytic carbonylation reactions, namely hydroformylation and hydroalkoxycarbonylation that time [17,18,19,20,21,22,23,24,25,26,27,28,29,30,31,32]. In the early 1990s, his attention turned also towards cross coupling reactions [33] and his first paper on carbonylative coupling was soon published. After relocating to the University of Pécs, one of his major field of interest was the aminocarbonylation of a wide variety of iodoalkenes and iodoarenes.

The goal of this paper is to review all the works on carbonylative coupling reactions reported by Professor Kollár as well as those studies published by researchers working in close collaboration with him in Hungary.

## 2. Formation of the Active Catalyst

**Figure 3:**
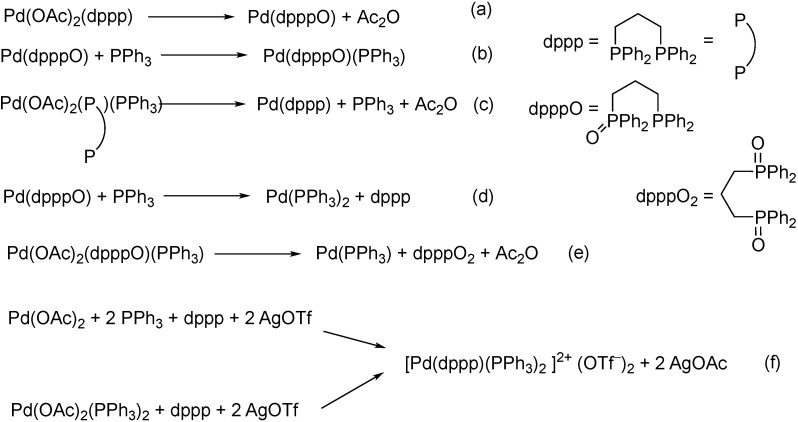
Formation of Pd(0) species from Pd(II) precursors (**a**–**e**), and that of the cationic Pd(II) complex in the presence of diphosphine, monophosphine and silver triflate (**f**).

The active catalysts of most coupling reactions are Pd(0) compounds. One way to the low-valent Pd(0) complexes is the employment of Pd(PPh_3_)_4_ or Pd_2_(dba)_3_ as precursors; the other is the reduction of Pd(II) salts in the presence of phosphine or other donor ligands. The usege of Pd(PPh_3_)_4_ may seem straitforward, however, its air sensibility might be a drawback for industrial applications. ${}^{31}$P NMR studies revealed that both monodentate (PPh_3_) and bidentate (dppp=1,3-bis-(diphenylphosphino)propane) phosphines are capable of reducing Pd(OAc)_2_ to zerovalent palladium resulting in also triphenylphosphine oxide or dppp hemioxide and dioxide (Figure 3a–e). When both dppp and PPh_3_ were present, also in the presence of silver triflate, the formation of the cationic square-planar complex [Pd(dppp)(PPh_3_)_2_]^+^ was reported without any reduction of Pd(II) (Figure 3f) [34].

The formation mechanism of the catalytically active species is expected to be more complicated in carbon monoxide atmosphere as CO competes with phophines for the available sites on the metal. The dissociation sequence as well as the PPh_3_/CO exchange reaction were examined via DFT calculations for the coordinatively saturated and all the unsaturated cases for Pd(0). For the 12–16e complexes, the oxidative addition of iodobenzene was compared and species Pd(PPh_3_)_2_ and Pd(PPh_3_)(CO) were found as the mostly preferred candidates kinetically [35].

## 3. Reactions in Conventional Solvents

**Figure 4:**
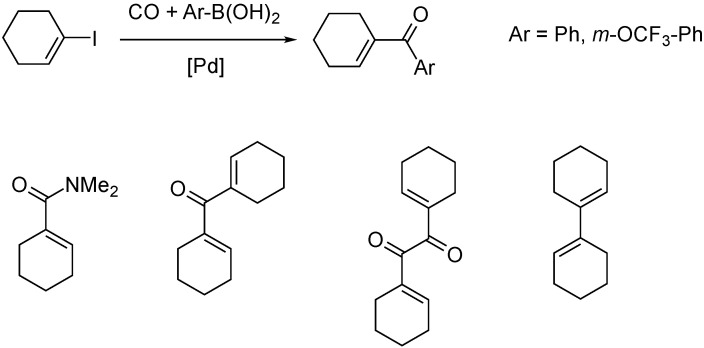
Carbonylative Suzuki-Miyaura reaction and its products.

The carbonylative Suzuki-coupling of iodocyclohexene with phenylboronic acid and 3-trifluoromethoxy-phenylboronic acid was investigated in the presence of Pd catalysts, in dimethylformamide solvent (Figure 4). Interestingly, DMF acted as nucleophile, resulting in primary carboxamide, proved by GC-MS measurements. Apart from the major product, various byproducts were detected, such as the (non-carbonylative) Suzuki-product, and the homocoupled cyclohexene dimers, as well as its keton and diketon derivatives [36]. The chemoselectivity towards cyclohexenyl-phenyl ketone reached 100% when DPPF (1,1′-bis(diphenylphosphino)ferrocene) ligand and DMSO solvent were employed. In the presence of DPPB (1,4-bis(diphenylphosphino)butane) and PPh_3_, the non-carbonylative coupling was dominant [37].

In a two-step reaction sequence, various primary amides and ketoamides were prepared. The first step involved palladium-catalyzed aminocarbonylation of iodoalkene and iodoarene substrate with tert-butylamide. The resulting amides and ketoamides were reacted with tert-butyldimethylsilyl triflate (TBDMSOTf) for the cleavage of the *^t^*Bu group, affording primary amides [38].

Ortho-alkoxy aryl iodides (2-iodoanisole, 5-chloro-7-iodo-8-methoxy-quinoline, and 5-chloro-7-iodo-8-benzyloxy-quinoline) were aminocarbonylated in the presence of in situ produced Pd(0) catalysts and simple primary and secondary amines. The chemoselectivity was shifted towards the formation of ketoamides when the CO pressure was increased to 40 bar [39].

**Figure 5:**
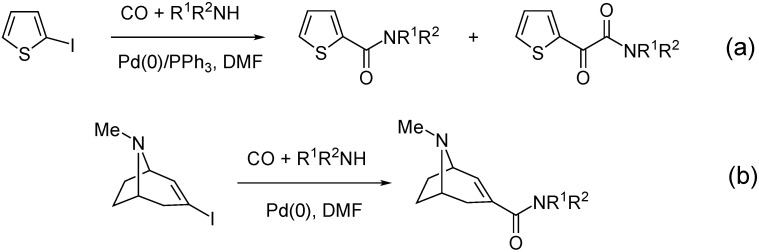
Aminocarbonylation of 2-iodotiophenes (**a**) and tropenes (**b**).

2-Iodothiophene was aminocarbonylated with simple primary and secondary amines as well as with amino acid esters as *N*-nucleophiles in the presence of Pd(0)/PPh_3_ catalysts. Atmospheric CO pressure resulted in practically zero conversion for the amino acid esters, however, at higher carbon monoxide pressure various ketocarboxamides, formed via double carbon monoxide insertion, were isolated with good yields (Figure 5a) [40]. Also high yield was achieved in the aminocarbonylation of tropenes. In this reaction, however, only the single CO insertion, that is, the formation of amides was observed (Figure 5b) [41].

N-acylated prolinates were synthesized from various iodoalkenes and iodoarenes reacting with methyl and benzyl prolinate. From iodobenzene and all the iodoalkenes only the formation of amides was observed, from iodonaphtalene a mixture of amides and ketoamides was obtained [42]. In the aminocarbonylation of various alkenyl and (hetero)aryl iodides tropane-based amines of biological importance were used, such as 8-azabicyclo[3.2.1]octan-3-one (nortropinone) and 3α-hydroxy-8-azabicyclo[3.2.1]octane (nortropine) as N-nucleophile. With iodoalkenes, the *N*-nucleophiles were selectively converted to the corresponding amide in the presence of Pd(0)/2 PPh_3_ catalysts. In the presence of iodo(hetero)arenes, the application of the bidentate Xantphos was necessary to eliminate the double carbonylation, thereby producing the target compounds selectively [43].

**Figure 6:**
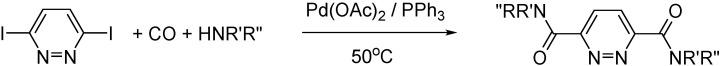
Pd-catalyzed aminocarbonylation of 3,6-diiodopyridazine with primary and secondary amines.

The Pd-catalyzed aminocarbonylation of 3,6-diiodopyridazine with several primary and secondary amines (including amino acid esters) resulted in 3,6-diamides in moderate to high yield in chemoselective reactions. The lack of double carbonylation product was explained by the close proximity of the aromatic ring nitrogen to the iodo substituent (Figure 6) [44].

**Figure 7:**
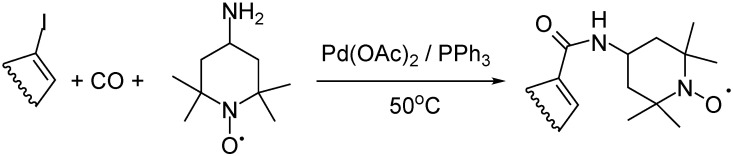
Pd-catalyzed aminocarbonylation of iodoalkenes with 4-amino-TEMPO.

The reactions of iodobenzene and iodoalkenes such as 1-iodocyclohexene, 4-tert-butyl-1-iodocyclohexene, α-iodostyrene, and 17-iodoandrost-16-ene with the free radical 4-amino-TEMPO afforded amides and ketoamides for iodobenzene and selectively amides for all the other cases. The free radical was partially reduced under aminocarbonylation conditions; however, the pure carbonylated products with a stable radical moiety were obtained after isolation (Figure 7) [45].

**Figure 8:**
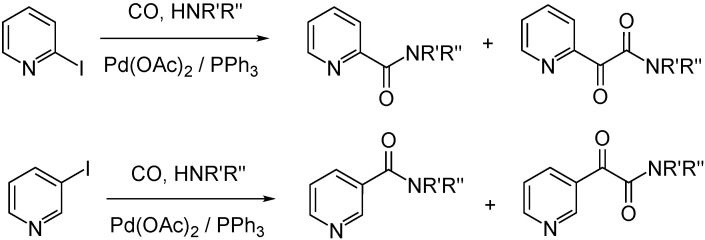
Pd-catalyzed aminocarbonylation of 2-iodopyridine and 3-iodopyridine with primary and secondary amines.

In palladium-catalyzed aminocarbonylation of 2-iodopyridine, 3-iodopyridine and iodopyrazine were coupled with CO and various primary and secondary amines. The biologically relevant N-substituted nicotinamides and 3-pyridyl-glyoxylamides were obtained from 3-iodopyridine as a result of simple and double carbon monoxide insertions, respectively. The chemoselectivity towards the ketoamide can be increased by the elevation of CO pressure. On the other hand, N-alkyl and N-aryl-carboxamides were obtained exclusively from CO pressure of 1 to 90 bar by using 2-iodopyridine and iodopyrazine (Figure 8) [46].

**Figure 9:**
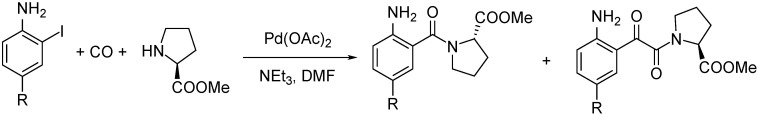
Pd-catalyzed aminocarbonylation of 2-iodoaniline in the presence of proline methylester.

2-Iodoaniline derivatives were employed as bifunctional substrates in palladium-catalysed carbonylation. Depending on the substituents of the iodoaromatic compounds, two types of species were prepared. With methyl or hydrogen in 4-position, 2-aryl-benzo[d][1,3]oxazin-4-one derivatives were formed. On the other hand, chloro, bromo, cyano or nitro groups in the same position resulted in the formation of dibenzo[b,f][1,5]-diazocine-6,12-dione derivatives. In the presence of various primary and secondary amines, such as tert-butylamine and amino acid methyl esters, as *N*-nucleophiles 2-ketoamides were obtained as major products in aminocarbonylation reaction with formal double carbon monoxide insertion (Figure 9) [47].

Reactive iodoalkenes, such as α-iodostyrene and α,$\alpha^{\prime }$-diiodo-1,4-divinylbenzene were prepared and introduced into Pd-catalyzed aminocarbonylation. With all the *N*-nucleophiles only single CO insertion was observed, that is, N-substituted phenylacrylamid products were obtained chemoselectively [48].

1,8-Diiodo-naphthalene was aminocarbonylated with various primary and secondary amines in the presence of in situ formed Pd(0)/PPh_3_ complexes. With primary amines, tert-butylamine, aniline, and benzylamine, the corresponding carboxamides were obtained in trace amounts as the main products were the N-substituted 1,8-naphtalinimides formed in ring closure [49].

In the Pd-catalyzed aminocarbonylation of (*E*)- and (*Z*)-1-iodo-1-dodecene odd-number carboxamides were synthesized in moderate to good yields, depending on the *N*-nucleophiles. As side products, amides with triple bond in the 2-position were also formed. It was assumed that the formation of 2-yn carboxamides took place via an iodo-alkenyl-palladium intermediate with a terminal carbonyl ligand [50].

Two highly reactive iodoalkenes, that is 1-iodo-1-(2-naphthyl)ethene and 1-iodo-1-(1-naphthyl)ethene) were prepared and used as substrates in Pd-catalyzed aminocarbonylation with various *N*-nucleophiles. The corresponding N-substituted naphthylacrylamides were produced chemoselectively in nearly quantitative yields [51].

**Figure 10:**
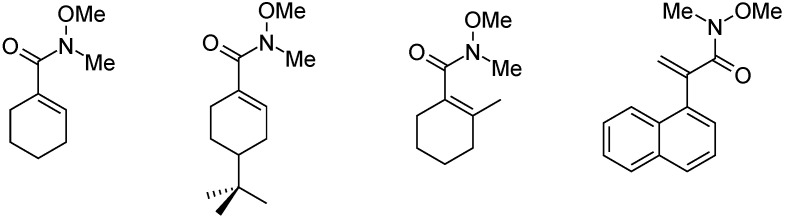
Weinreb amides from the aminocarbonylation of the corresponding iodoalkenes.

Weinreb amides were prepared in high isolated yield from iodoarenes and iodoalkenes in aminocarbonylation reactions with N,O-dimethylhydroxylamine. With exception of 2-iodotiophene, no ketoamides were produced, even at higher (60 bar) CO pressure. The highest isolated yield (85%) was achieved for 1-iodo-2-methylcyclohexene (Figure 10) [52].

2-Iodobenzyl bromide was reacted with various *N*-nucleophiles and the resulting 2-iodobenzylamines were aminocarbonylated in the presence of Pd complexes. The intramolecular reaction proved to be highly chemoselective leading to 1-isoindolinone derivatives in high yields [53]. 5-Carboxamido-7-iodo-8-benzyloxyquinolines were synthesized in Pd-catalyzed aminocarbonylation of 5,7-diiodo-8-benzyloxyquinoline with high yield. The reaction proceeded with high regioselectivity leading to 5-carboxamido derivatives, that is, the 7-iodoaryl functionality remained untouched. The iodoarene functionality of the target carboxamides was suitable for further functionalization [54].

1,2,3,4-Tetrahydrophthalazin-1-one and 1,2,3,4-tetrahydrophthalazin-1,4-dione derivatives were synthesized in Pd-catalyzed hydrazinocarbonylation of 2-iodobenzyl bromide and 1,2-diiodobenzene as bifunctional substrates. The reaction with the latter initial compound proved to be less selective with the formation of various hydrazide side products [55].

5-Iodo- and 4,5-dibromo-2-methylpyridazin-3(2H)-ones were successfully converted to the corresponding amides with high chemoselectivity with most of the *N*-nucleophiles. The dibromo substrate showed high reactivity with primary amines. With secondary amines, however, the formation of aminosubstituted bromopyridazinones dominated, that is, the C-N coupling took place without the involvement of carbon monoxide [56].

**Figure 11:**
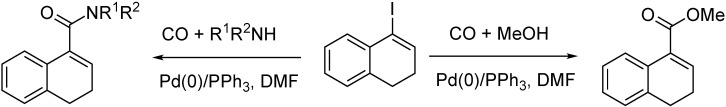
Pd-catalyzed aminocarbonylation of 1-iodo-3,4-dihydronaphtalene.

1-Iodo-3,4-dihydronaphtalene was carbonylated in the presence of Pd-phosphine systems. For both aminocarbonylation and alkoxycarbonylation very high isolated yields (up to 96%) were achieved resulting in 1-carboxamido-3,4-dihidronaphtalenes and 1-methoxycarbonyl-3,4-dihydronaphtalene, respectively (Figure 11) [57].

The aminocarbonylation of iodobenzene, 1-iodocyclohexene and 1′-iodostyrene in the presence of *N*-nucleophiles containing pyridyl moieties (2-, 3- and 4-picolylamine, N-ethyl-4-picolylamine, di-(2-picolyl)amine) was investigated. From iodobenzene a mixture of amides and ketoamides was obtained with the predominant formation of the ketoamides, in most cases. The chemoselectivity towards the doubly carbonylated product reached 94% at 40 bar of CO pressure when 3-picolylamine was employed as N-nucleophile. No double carbonylation was observed, however, for the iodoalkene substrates [58]. Similar dependence upon the substrate was observed when iodopyridine model compounds were aminocarbonylated with various primary and secondary amines [59]. In the aminocarbonylation reaction of iodocamphene and steroidal iodoalkenes in the presence of picolylamines N-picolylcarboxamides were produced. The iodoalkenes were synthesized from the corresponding ketones, that were converted to hydrazones and reacted further with iodine in the presence of base [60]. Mixed products were obtained as well in the aminocarbonylation of diiodopyridines. For orto-diiodo compounds (2,3-diiodopyridine and 2-Cl-3,4-diiodopyridine) imides were also formed [61]. With iodouracil derivatives, higher selectivities towards ketoamides were achieved when 40 bar of CO pressure was used. For the weaker N-donor aniline, however, no double carbonylation was reported [62]. The aminocarbonylation of medium-sized 3-aminolactams as *N*-nucleophiles resulted in a mixture of amides and ketoamides with the ratio strongly depending on the substrate. When PPh_3_ was replaced by xantphos, as an ancillary ligand, the exclusive formation of amides was reported, without the traces of double carbonylation products [63].

The employment of hydrazines as *N*-nucleophiles opens the possibility to produce hydrazides. Functionalization of 17-iodo and bromo substituted androst-16-ene derivatives in hydrazinocarbonylation reaction resulted in 17-(N-phenylaminocarbamoyl)-, 17-(N-diphenylaminocarbamoyl)-, 17-(N-amino-N-methylcarbamoyl)-, and 17-(N-(dimethylamino) carbamoyl)androst-16-ene products in high isolated yields, mostly with excellent regioselectivity [64,65].

### 3.1. Preparation of Carbonyl Compounds with Steroid Scaffolds

Both 17-iodo-16-ene and 6-iodo-5-ene functionalities of androstane derivatives were reacted with vinyltributyltin in Stille reactions. The resulting iodo-vinyl derivatives could be converted in high yield to methylester in a hydromethoxycarbonylation reaction, or to a carboxamide in the presence of CO and piperidine [66].

**Figure 12:**
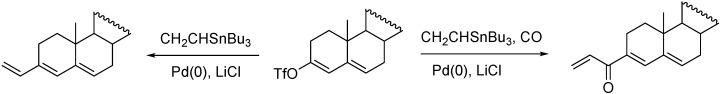
Pd-catalyzed vinylation and carbonylative vinylation of steroidal triflates.

Steroid 2-enyl- and 3,5-dienyl-3 triflates and estrone-3-triflate were vinylated with vinyltributylstannane in the presence of Pd(0) catalysts. In CO atmosphere, unsaturated ketones were obtained in chemoselective reactions with high yield (Figure 12) [16].

**Figure 13:**
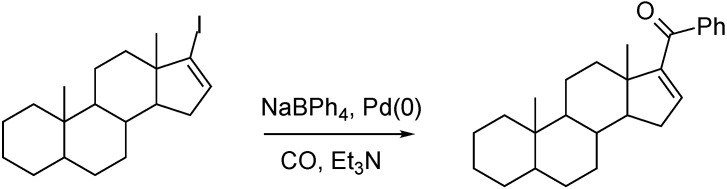
Pd-catalyzed carbonylation of 17-iodo-androst-16-ene in the presence of NaBPh_4_.

Steroidal phenyl ketones were prepared in high yields by palladium-catalyzed carbonylation reactions of 17-iodo-androst-16-ene derivatives under mild reaction conditions, with NaBPh_4_ as phenylating agent. Alkenyl bromides or enol triflates resulted in lower yields as compared to the alkenyl iodides (Figure 13) [67].

**Figure 14:**
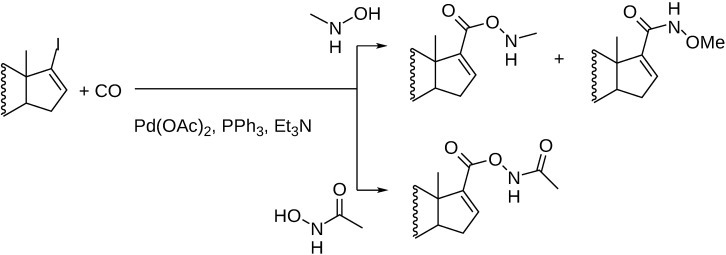
Formation of hydroxamic acid derivatives in Pd-catalyzed carbonylation reactions.

Hydroxamic acid derivatives were synthesized from 17-iodo-androst-16-ene based substrates. Depending on the substrate both O-acylation and N-acylation took place; O-methyl-hydroxylamine afforded the 17-(N-methoxy-carbamoyl) structures, acetohydroxamic acid led to O-acylation exclusively, whereas the carbonylation of N-methyl-hydroxylamine resulted in both the O-acylated and the N-acylated products (Figure 14) [68].

The Pd-catalyzed hydrazinocarbonylation of some steroid derivatives possessing iodo-alkenyl moiety were carried out in the presence of a base and acetic or benzoic hydrazide as the nucleophilic reagent. The corresponding N-acetamido-carbamoyl or N-benzamido-carbamoyl derivatives were obtained in high yields. Some of these products served as starting materials for the synthesis of new steroidal 1,3,4-oxadiazole compounds [69].

Using hydroxylamines as *N*-nucleophiles resulted in a yet another strongly related reaction. Various steroidal hydroxamic acid derivatives were synthesized from the corresponding iodo-alkenyl or enol triflate derivatives. In principle, the Pd-acyl intermediate, formed by the oxidative addition of the substrate followed by CO insertion, can react with either the NH or the OH functionality of the hydroxylamine derivative. The electron withdrawing R substituent of hydroxylamines RNH-OH resulted in O-acylation, whereas N-acylation was dominated in most cases with the hydroxylamine CH_3_NH-OH. Interestingly the steric hindrance of the bulky ^*t*^Bu substituent suppressed the rate of N-acylation thereby making the O-acylation products dominant [70].

17-Alkoxycarbonyl- and 17-carboxamido-13α-estra-1,3,5(10),16-tetraenes were synthesized in alkoxycarbonylation and aminocarbonylation reactions, respectively, from the corresponding iodo derivative, which was obtained from the keto derivative converted to hydrozone in the first step. The alkoxycarbonylation resulted in the methylester with acceptable yield. 17-Carboxamides were obtained in better yields with a range of *N*-nucleophiles [71]. In similar reactions, 17-alkoxycarbonyl- and 17-carboxamido-3β-hydroxy-13α-androsta-5,16-diene derivatives were synthetized in high yields from the corresponding β-hydroxy-17-iodo-13α-androsta-5,16-diene. Using water as O-nucleophile resulted in the 17-carboxylic acid derivative via a hydroxycarbonylation reaction [72]. 17a-Methoxycarbonyl- and 17a-carboxamido-d-homoestra-1,3,5(10),17-tetraene derivatives were also prepared from the corresponding 17a-iodo-d-homoestra1,3,5(10),17-tetraene derivatives using methanol and primary amines as well as secondary amines as O- and *N*-nucleophiles, respectively. Both the natural (13β) and the epi (13α) series of compounds were isolated. Elevating the CO pressure up to 40 bar resulted in excellent yields even for the less reactive 13α compounds [73]. Steroids containing both the 17-iodo-16-ene and 3-iodo-3,5-diene structural motifs were converted to 3,17-dicarboxamido-androst-3,5,16-triene derivatives in aminocarbonylation reaction. For *N*-nucleophiles, tert-butylamine, piperidine and methyl alaninate were used [74].

**Figure 15:**
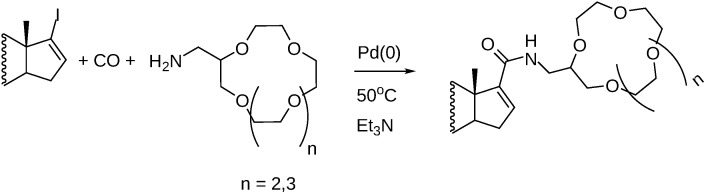
Preparation of steroidal crown ethers.

17-Iodo-androsta-16-enes were employed as substrates in aminocarbonylation with crown-ethers possessing an aminomethyl group (Figure 15). Excellent yields were reported regardless of the size of the crown-ether moiety [75].

**Figure 16:**
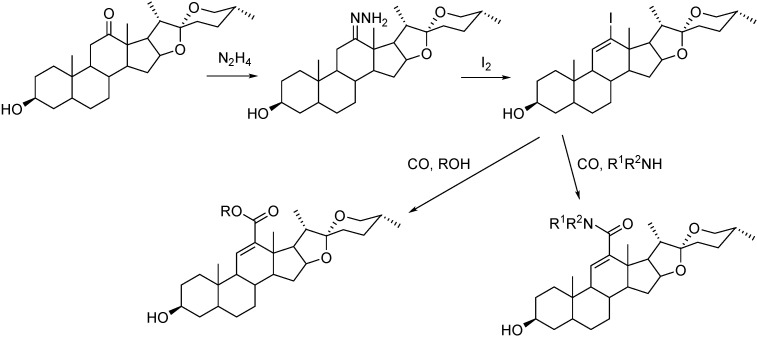
Synthesis of 12-carboxamido- and 12-carboxyl-11-spirostenes.

In Pd-catalyzed carbonylation reactions,12-carboxamido- and 12-carboxyl-11-spirostenes were synthesized from the corresponding 12-iodo-11-ene derivative under mild conditions (Figure 16). The preparation of the iodo-alkene substrate is based on the conversion of the 12-keto derivative (hecogenin) to hydrazone, which was treated with iodine in the presence of a base. While various 12-carboxamides were produced via aminocarbonylation in moderate to high yields by using simple alkyl/arylamines or amino acid methylesters as *N*-nucleophiles, low yields were achieved with alcohols as O-nucleophiles [76]. The preparation of the iodoalkenes followed a similar methodology for 11-iodo-androst-4,9(11)-diene which was converted to the corresponding carboxamides with simple alkyl/arylamines or amino acid methylesters as *N*-nucleophiles in high yield [77].

**Figure 17:**
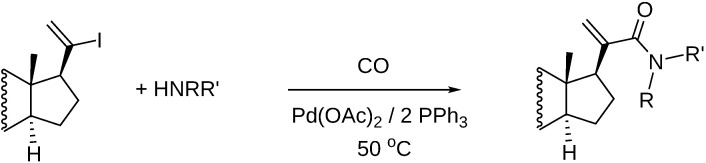
Aminocarbonylation of iodoalkenes affording 20-carboxamides.

20-Carboxamidopregnene derivatives, such as 3β-acetoxy-5α-pregn-20-ene-20- carboxamides and 5α-pregn-20-ene-20-carboxamides were synthesized from the corresponding iodoalkenes and various *N*-nucleophiles (Figure 17). The iodoalkenes were produced in multistep reactions, namely selective hydrogenation, the reaction of the subsequent ketones with hydrazine, and the conversion of the hydrazones to iodoalkenes with iodine [78].

17-Formyl-androst-16-ene and its analogues were synthesized from the corresponding 17-iodo-16-ene derivatives in Pd-catalyzed formylation reactions in the presence of CO. Tributyltin hydride served as a hydrogen source. The formation of side products (androst-16-ene and androst-15-ene derivatives) were also reported; the maximal chemoselectivity was achieved with dppb (1,4-diphenylphosphynobutane) as ligand [79].

### 3.2. Ferrocene-Based Substrates

**Figure 18:**
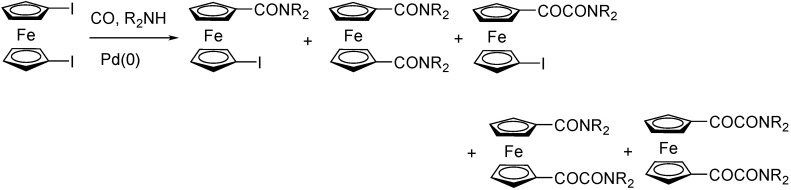
Aminocarbonylation of diiodoferrocene.

The Pd-catalyzed aminocarbonylation of iodoferrocene led to the mixture of amides and ketoamides. With methyl glycinate, as N-nucleophile, mostly amides were formed in the presence of Et_3_N. With DBU (1,8-diazabicyclo[5.4.0]undec-7-ene), the ratio of ketoamides increased, however, formation of side products were also observed [80]. With 1,1′-diiodoferrocene, apart from the symmetric amides and ketoamides, mixed amide/ketoamide products and partially unreacted derivatives were observed (Figure 18) [81]. With stepwise reaction, it was also possible to form two different carbamoyl groups on the two rings of ferrocene, e.g. diamide with one *^n^*Bu and one morpholino moieties. The two amido groups were stabilized by intramolecular hydrogen bonds [82].

Using 40 bar CO pressure, high chemoselectivity towards the ketoamides could be achieved with morpholine and 3,5-dimethyl-piperidine as secondary amines. Further increase of the pressure did not increase the ratio of the double carbonylated products [83]. Somewhat lower chemoselectivity was achieved with piperidine, diethylamine, and *^n^*Bu_2_NH [84]. Various unsymmetrical disubstituted ferrocenoyl amino acids were produced by Pd-catalyzed aminocarbonylation starting from 1,1′-diiodoferrocene in one-pot reactions. All the products adopted ordered structures stabilized by intramolecular H-bonds [85].

### 3.3. Carbonylation on Macromolecular Cavitand Scaffolds

Carbonylation reactions can also be successfully applied to various macromolecules as substrates. These macromolecules include cavitands, which are bowl-shaped or tubular molecules and possess well-formed large hydrophobic cavities. Generally, cavitands are prepared by organic chemical synthetic methods and very few publications can be found in the literature dealing with homogeneous catalytic syntheses on cavitand scaffolds. Moreover, carbonylative syntheses applied on a cavitand skeletons were not mentioned in the literature at all, with the exception of the works of our research group. In the recent ten years, our research group had developed several palladium- and copper-catalyzed reactions on a cavitand sceleton [86,87,88,89], including palladium-catalyzed aminocarbonylation [90,91,92,93].

**Figure 19:**
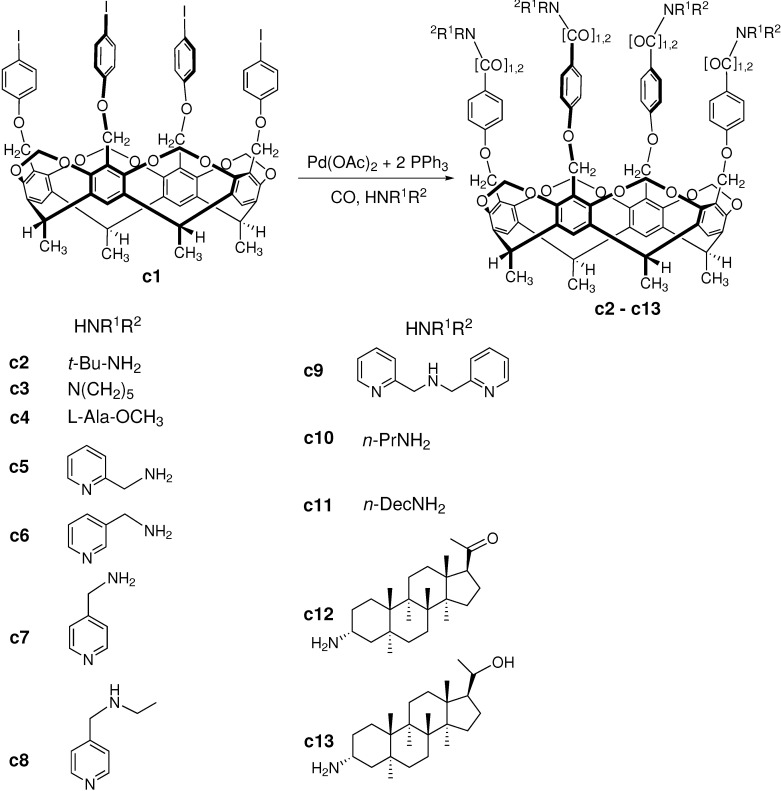
Scheme of the aminocarbonylation reaction on cavitand scaffold.

The **c1** tetraiodo-cavitand compound, bearing four excellent leaving groups, is a great substrate for different homogeneous catalytic cross-coupling reactions including palladium-catalyzed aminocarbonylation. The **c1** cavitand substrate was synthesized in a four-step consecutive reaction sequence and the aminocarbonylation reaction was proved to be highly efficient and selective synthetic tool for the synthesis of extended cavitands, decorated with varied amide groups on the upper rim (Figure 19). The aminocarbonylation reactions were performed at atmospheric and high pressure also in the presence of in situ prepared Pd(0) catalyst at moderate temperature (50–60 °C).

Besides primary (*tert*-butyl-amine (for **c2**) [90], 2-, 3-, 4-picolylamine (**c5**, **c6**, **c7**) [91], *n*-propylamine (**c10**) [94], *n*-decylamine (**c11**) [94]) and secondary amines (piperidine (**c3**) [90], 4-(ethylaminomethyl)pyridine (**c8**) [91], di-(2-picolyl)amine (**c9**) [91]), a chiral amino acid (L-alanine methyl ester hydrochloride (**c4**) [90]) and aminosteroids (3α-amino-5α-pregnan-20-one (**c12**), 3α-amino-5α-pregnan-20-ol (**c13**) [92]) were employed as *N*-nucleophile for the preparation of the novel tetracarboxamido and tetraketocarboxamido cavitands. In general, at atmospheric pressure the formation of carboxamide product was favorable while the higher CO pressure (30–90 bar) generated a superior chemoselectivity towards the ketocarboxamides. Furthermore, increasing both the pressure and the molar ratio of the amine resulted in an increase in selectivity toward a tetraketocarboxamido cavitand products but in some cases, high CO pressure (60–90 bar) inhibited the reaction [91] and no product was formed at all. It has been proved also that bases such as triethylamine, pyridine, DBU and potassium carbonate thoroughly influenced the chemoselectivity towards carboxamides/ketocarboxamides [94].

It is worth mentioning that when the molar equivalents of the amine reactants were decreased below 4, that is, less than a stoichiometric amount of the *N*-nucleophile was used, only the tetrafunctionalized products could be isolated along with unreacted starting tetraiodocavitand (**c1**) and neither the formation of mono-, di- or trifunctionalized products nor that of the mixed-substituted carboxamido- or ketocarboxamido-cavitands was observed [90,91]. The formation of mixed-substituted product was not observed also when two different ‘competing’ amines were applied during the reaction [94]. Using two amines in the aminocarbonylation reaction, either separately or together, four products were obtained only: the two carboxamide type products and the two ketocarboxamide type products (**c10**, **c11** with 1 or 2 CO inserted) bearing four identical functionalities at the upper rim.

To introduce chiral moiety on the upper rim, L-alanine methyl ester hydrochloride [90] and aminosteroids (3α-amino-5α-pregnan-20-one and 3α-amino-5α-pregnan-20-ol) [92] were used as *N*-nucleophiles during the aminocarbonylation. The macromolecules produced this way can serve as excellent chiral selectors.

**Figure 20:**
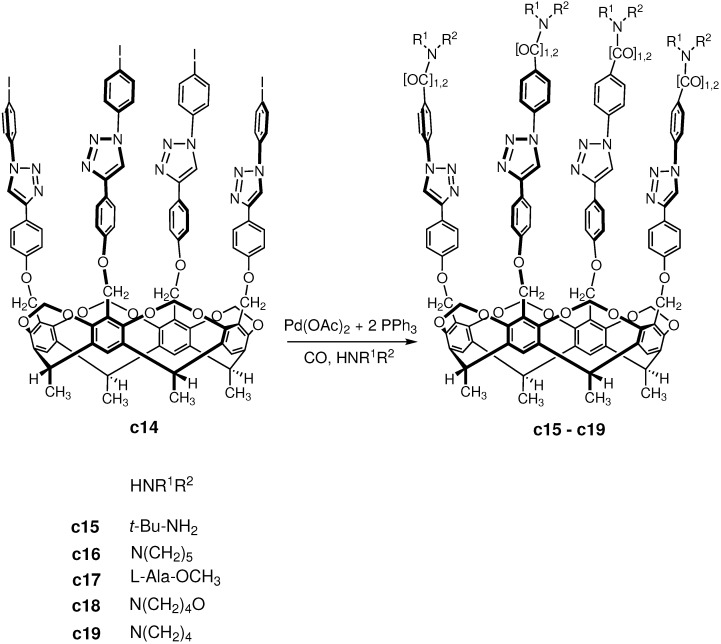
Scheme of the aminocarbonylation reaction on deepened cavitand scaffold.

The **c14** deepend cavitand bearing also four iodoaryl groups on the upper rim is even larger than **c1** cavitand. Despite the molar mass of **c14** is above 2000 g/mol hence both solubility and steric problems could have been expected during the reaction, **c14** was also suitable as substrate for aminocarbonylation [93] and successful reactions were performed with five different amines (Figure 20) at atmospheric and high pressure (90 bar) also.

In agreement with the previous results, the high carbon monoxide pressure was favorable for the formation of the ketocarboxamide products, while at atmospheric pressure the carboxamide products were formed in larger amounts. The best chemoselectivity was observed with *tert*-butylamine at high-pressure, and good chemoselectivity was achieved also with *tert*-butylamine, L-alanine methyl ester and pyrrolidine at atmospheric pressure, moreover, with piperidine at high pressure.

## 4. Reactions in Biomass-Based Solvents

Solvents are intrinsic part of many chemical reactions and their properties such as acidity/basicity, polarity, viscosity, density, dielectric constant etc. primarily affect the efficiency of chemical transformations [95]. Consequently, the “solvent friendly chemical thinking” has evolved due to many advantages in both laboratory and industrial operations. However, the industrial activities involving solvents result in the release of volatile organic compounds (VOCs) including conventional solvents into the environment for example for EU27 over 7 million tons annually. On the other hand, the common organic solvents including typical reaction media used in carbonylation reactions such as benzene, toluene, DMF, DMA, dioxane or acetonitrile are toxic, non-renewable crude oil derivatives. Thus, the replacement of conventional organic solvents with green alternatives having low vapour pressure, low flammability, low toxicity, and limited negative impacts on the environment is a key challenge in the development of greener and cleaner chemical technologies [96].

Although, “solvent free” transformation could offer environmentally friendly solutions, many thousands if not millions of reactions can only be operated in the presence of solvents as auxiliary materials. Recently it was demonstrated the gamma-valerolactone (GVL), which has been considered as a renewable platform molecule [97] and can be produced from lignocellulosic biomass [98,99], can be used an alternative reaction medium for homogeneous [100,101,102,103] catalysis involving Pd-catalyzed carbonylation of iodoaromatic compounds in the presence of N- or O-nucleophiles.

**Figure 21:**
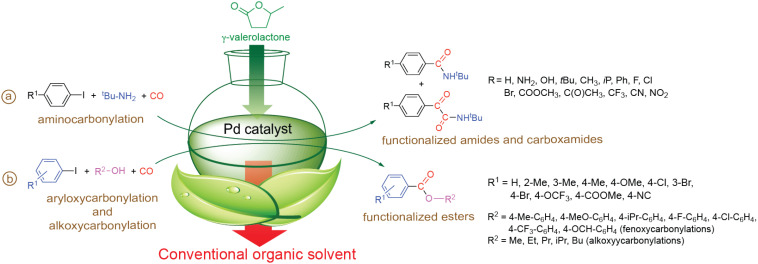
Conversion of iodoaromatic compounds in GVL.

It was demonstrated that various 4-substituted iodoaromatic compounds could be converted to correspondig carboxamides and ketocarboxamides in GVL (Figure 21a). In comparison with conventional solvent such as DMF, a slightly lower activity and comparable selectivity of widespred used Pd/PPh_3_ catalyst system was demonstrated under 1 bar of CO at 50 °C for 24 h. A significant CO pressure dependent selectivity was observed in the pressure range of CO 1–25 bar. While outstandig selectivity towards ketocarboxamides were shown over 25 bar, the activity of the catalytic system decreased dramatically. The optimum pressure and temperature were found to be 25 bar and 50 °C, respectively. Both conversion and selectivity towards karboxamides were significantly affected by electronic properties of para substituents [102].

## 5. Carbonylation Reactions in Ionic Liquid and on Supported Ionic Liquid Phase (SILP)

Steroids with 17-iodo-16-ene functionality were catalytically converted to amides with morpholine and Pd(0)-phosphine complexes in ionic liquid media. Highest conversion in the fifth run was achieved with PPh_3_ as ancillary ligand and [bmim]${}^{+}$[BF_4_]${}^{-}$ as solvent (bmim = 1-butyl-3-methylimidazolium) [104]. 1-Iodo-cyclohexene and 17-iodo-androst-16-ene were converted to the corresponding carboxamide derivatives in excellent yields with amino acid methyl esters as *N*-nucleophiles in [bmim]${}^{+}$[BF_4_]${}^{-}$ and [bmim]${}^{+}$[BF_4_]${}^{-}$. Under the same conditions, ketoamides were produced with the iodobenzene substrate. The reaction was repeated several times with the same ionic liquid–catalyst mixtures with only a small loss of catalytic activity [105]. The steroid-amino acid conjugates were reacted with N,N′-dicyclohexyl-carbodiimide (DCC) resulting in a mixture of imides and N-acylureas [106].

**Figure 22:**
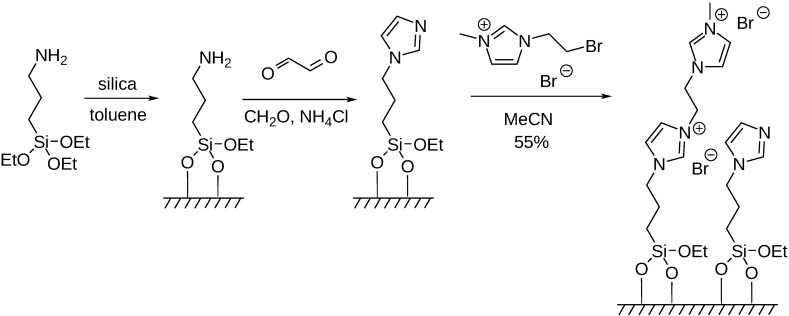
Preparation of the SLIP phase used for immobilizing the Pd catalyst.

Silica modified with organic dicationic moieties proved to be an excellent support for palladium catalysts used in the aminocarbonylation of aryl iodides. By an appropriate choice of the reaction conditions, the same catalyst could be used for selective mono- or double carbonylations leading to amide and α-ketoamide products, respectively. The best catalyst could be recycled for at least 10 consecutive runs with a loss of palladium below the detection limit [107]. The efficiency of a palladium catalyst, immobilized on a supported ionic liquid phase (SILP) with adsorbed 1-butyl-4-methylpyridinium chloride (Figure 22), was scrutinized in aminocarbonylation reactions. Double carbonylation was found to be the major reaction using different iodoarenes and aliphatic amines as substrates.

Application of aniline derivatives as nucleophiles led to the exclusive formation of substituted benzamides. The stabilisation effect of the adsorbed pyridinium ionic liquid was compared to that of imidazolium and phosphonium derivatives. It was proved that the pyridinium SILP-palladium catalyst could be reused in at least 10 cycles [108].

The application of palladium catalysts supported on phosphonium ion modified silica was scrutinized in aminocarbonylation reactions of aryl iodides. In contrast to catalysts immobilized on supports decorated with imidazolium ions, the application of phosphonium type supported ionic liquid phases facilitated to carry out double carbonylation with good selectivity in apolar toluene, leading to a considerable decrease in the amount of leached palladium. An even better stabilization of the palladium catalyst was achieved by introducing dicationic organic moieties incorporating both imidazolium and phosphonium ions on the surface of the silica support. The former catalyst, obtained from the supported phosphonium ionic liquid phase, was found to be superior in monocarbonylations. The amide products were obtained in good yields by the careful choice of the reaction conditions, such as reaction temperature and pressure and by the appropriate selection of the base [109]. A new SILP (Supported Ionic Liquid Phase) palladium catalyst was prepared and characterized by ${}^{13}$C and ${}^{29}$Si CP MAS NMR, differential thermogravimetry, FT-IR and transmission electron microscopy. The presence of the grafted pyridinium cations on the surface of the support resulted in the formation of highly dispersed Pd nanoparticles with their diameter in the range of 1–2 nm. Apart from the aminocarbonylation reaction, the catalyst was also suitable for the synthesis of active pharmaceutical ingredients. Catalyst recycling and palladium leaching studies were carried out leading to CX-546(1-(1,4-benzodioxan-6-ylcarbonyl)piperidine), Moclobemide, Nikethamide and a precursor of Finasteride. The latter reaction proved that apart from aryl iodides, iodoalkenes can also be converted into the products with the help of the heterogeneous catalyst. The palladium loss was observed to depend considerably on the nature of the reaction partners [110].

## 6. Concluding Remarks

This review attempted to compile the advances in carbonylative coupling reactions made by Professor Kollár and co-workers in the last almost three decades. As it was demonstrated, these reactions serve as powerful tools for the conversion of a very wide variety of aryl/alkenyl halides or halide equivalents, such as triflates, to the corresponding carbonyl compounds and carboxylic acid derivatives. The immobilization and thereby the reusability of the catalysts may open also a way towards industrial applications. As the demand for selective synthetic methods aiming at the preparation of fine chemicals steadily increases, new achievements are expected employing new ligands and/or environmentally more benign catalytic systems.
